# MUBII-TB-DB: a database of mutations associated with antibiotic resistance in *Mycobacterium tuberculosis*

**DOI:** 10.1186/1471-2105-15-107

**Published:** 2014-04-14

**Authors:** Jean-Pierre Flandrois, Gérard Lina, Oana Dumitrescu

**Affiliations:** 1 Laboratoire de Biométrie et Biologie Évolutive, UMR CNRS 5558, Université Claude Bernard – Lyon 1, 43 bd. du 11 Novembre 1918, 69622 Villeurbanne Cedex, France; 2 CIRI, International Center for Infectiology Research, LabEx Ecofect, Université Lyon1; Inserm, U1111, Ecole Normale Supérieure de Lyon; CNRS, UMR5308, 69000 Lyon, France; 3 Hospices Civils de Lyon, 69002 Lyon, France

**Keywords:** Tuberculosis, Antibiotics, Mutation database, Sequence database, Web

## Abstract

**Background:**

Tuberculosis is an infectious bacterial disease caused by *Mycobacterium tuberculosis*. It remains a major health threat, killing over one million people every year worldwide. An early antibiotic therapy is the basis of the treatment, and the emergence and spread of multidrug and extensively drug-resistant mutant strains raise significant challenges. As these bacteria grow very slowly, drug resistance mutations are currently detected using molecular biology techniques. Resistance mutations are identified by sequencing the resistance-linked genes followed by a comparison with the literature data. The only online database is the TB Drug Resistance Mutation database (TBDReaM database); however, it requires mutation detection before use, and its interrogation is complex due to its loose syntax and grammar.

**Description:**

The MUBII-TB-DB database is a simple, highly structured text-based database that contains a set of *Mycobacterium tuberculosis* mutations (DNA and proteins) occurring at seven loci: *rpo*B, *pnc*A, *kat*G; *mab*A(*fab*G1)-*inh*A, *gyr*A, *gyr*B, and *rrs*. Resistance mutation data were extracted after the systematic review of MEDLINE referenced publications before March 2013. MUBII analyzes the query sequence obtained by PCR-sequencing using two parallel strategies: i) a BLAST search against a set of previously reconstructed mutated sequences and ii) the alignment of the query sequences (DNA and its protein translation) with the wild-type sequences. The post-treatment includes the extraction of the aligned sequences together with their descriptors (position and nature of mutations). The whole procedure is performed using the internet. The results are graphs (alignments) and text (description of the mutation, therapeutic significance). The system is quick and easy to use, even for technicians without bioinformatics training.

**Conclusion:**

MUBII-TB-DB is a structured database of the mutations occurring at seven loci of major therapeutic value in tuberculosis management. Moreover, the system provides interpretation of the mutations in biological and therapeutic terms and can evolve by the addition of newly described mutations. Its goal is to provide easy and comprehensive access through a client–server model over the Web to an up-to-date database of mutations that lead to the resistance of *M. tuberculosis* to antibiotics.

## Background

Tuberculosis (TB) is an infectious disease caused by a slow-growing bacterium, *Mycobacterium tuberculosis*, which has been linked to humans since the beginning of the early human expansion from east Africa [1]. Tuberculosis was the main cause of deaths in Western Europe between the seventeenth century and the end of the nineteenth century [2]. It remains a major health threat, killing more than a million individuals every year worldwide. Although the WHO claims that its goal to halt and reverse the TB epidemic by 2015 has already been achieved and that the TB mortality rate has decreased by 41% since 1990, the global burden of TB remains. In 2011, there were an estimated 8.7 million new cases of TB, and 1.4 million people died from TB.

No fully effective vaccination is possible because the Bacille de Calmette et Guérin (BCG)-vaccine protection against TB varies among populations [3] and provides only limited protection during childhood [4]. The limitation of the TB expansion is thus based on the improvement and generalization of diagnostic methods [5] and early antibiotic therapy. The WHO recommends the following combined therapy employing first-line antibiotics: rifampicin (or rifabutin), isoniazid, pyrazinamide and ethambutol for two months followed by rifampicin and isoniazid for four months [6].

However, irregular and low-dose treatment has led to the emergence and spread of multidrug-resistant (MDR) and extensively drug-resistant (XDR) *Mycobacterium tuberculosis* complex (MTBC) strains that present significant challenges in disease control [6-8]. The resistance increases in high MDR-TB burden countries, where the number of cases was estimated to be 60,000 worldwide in 2011. In these countries, the proportion of resistant strains varies from 5 to 19% of new TB cases and up to 50% for retreatment cases. This increase in resistance has led to the promotion of laboratory antimicrobial testing for all isolates [6]. In the case of antibiotic resistance, the therapy is conducted using second-line drugs, such as aminoglycosides or non-conventional antibiotics (such as fluoroquinolones).

The traditional phenotypic drug susceptibility test induces serious delays in the detection of resistance due to the extremely slow growth of *M. tuberculosis*, the answer being obtained in most cases two weeks after the isolation of the bacteria. Another drawback of the *in vitro* susceptibility testing is the inadequate detection of resistance to new drugs and to pyrazinamide [9-11]. The rapid diagnosis of drug resistance by molecular methods is essential to initiate effective antibiotic therapies and to prevent the transmission of drug-resistant strains [12].

*M. tuberculosis* acquires drug resistance primarily through mutations in specific genes [13,14]. The mutations associated with drug resistance occur in *rpo*B for rifampicin (RIF), in *kat*G and the *mab*A *(fab*G1*)-inh*A operon for isoniazid (INH), in *pnc*A for pyrazinamide (PZA), in *rrs* for amikacin (AMK) and in *gyr*A and *gyr*B for fluoroquinolones (FQs) [13,15-17].

Several commercial molecular kits are available to detect *M. tuberculosis* resistance using line-probe assays or real-time PCR, and these kits allow for the prediction of drug resistance in clinical specimens within one working day [9]. They can detect the mutations responsible for resistance to RIF alone (GeneXpert MTB/RIF (Cepheid), INNO-LiPA® Rif. TB (Innogenetics)), to RIF + INH (GenoType® MTBDRplus (Hain LifeScience GmbH)), or to FQs + AMK (GenoType® MTBDRsl (Hain LifeScience GmbH)), but their sensitivity varies, depending on the antibiotic [18]. These kits, unfortunately, detect only the most frequent mutations [18,19]. An alternative approach that allows for the exhaustive detection of mutations consists of the PCR amplification and sequencing of resistance-linked genes [20,21] and, more experimentally, of complete genome sequencing [22]. It is, however, time consuming to compare the sequences with the reference genome and the mutation identity with the literature data. Recently, Sandgren *et al*. [23] established a comprehensive database that gathers information on the mutations associated with TB drug resistance and the frequency of the most common mutations associated with resistance to specific drugs. The TBDReaM database is a free online resource that allows for the molecular diagnosis of resistant TB after the processing of the amplified sequences by any method. Although very helpful, the TBDReaM database has not been updated since April 2010, and its usage remains time consuming because it demands prior processing of the TB nucleotide sequences to detect mutations and cannot be easily interrogated due to its relaxed grammatical conception.

Here, we describe MUBII-TB-DB, a database of the mutations of *M. tuberculosis* linked to resistance to first-line and second-line antibiotics that can be used to identify the mutations from a submitted sequence. This database and its related software, MUBII, have been developed to satisfy the need of clinical microbiology labs to easily analyze *M. tuberculosis* sequences and to link the results to a potential therapeutic failure. MUBII combines the use of reconstructed mutated gene sequences that can be searched by BLAST, aligned against the wild-type gene sequence, and compared with the mutation database. This concept can be easily adapted to the microbiological identification of other microbial mutations.

## Construction and contents

### Mutations database

#### *Data*

The database was constructed based on a systematic review of the literature, as described below. We focused on publications reporting an association between specific mutations in clinical isolates of *M. tuberculosis* and phenotypic resistance to INH, RIF, PZA, FQs, and AMK. The genes studied were *rpo*B for RIF; *inh*A, *kat*G*,* and the promoter region of the *mab*A*(Fab*G1*)-inh*A operon for INH; *pnc*A for PZA; *gyr*A and *gyr*B for FQs; and *rrs* for AMK. As a starting point, we used the TBDReaM database because it constitutes a comprehensive resource on drug resistance mutations in *M. tuberculosis* based on studies published from January 1966 to December 2009 [23]. Additional publications on *M. tuberculosis* resistance mutations recorded in MEDLINE from December 2009 to March 2013 were included in the analysis (see *infra*). All publications reporting mutations, including the ones recorded in the TBDReaM database, were carefully reviewed for the consistency of the information about the mutated nucleotide and amino acid. We used the codon numbering given by the annotation of the *M. tuberculosis* whole genome sequence published in [24].

### Strategy of the systematic literature review

All studies reporting an association between specific mutations in clinical isolates of *M. tuberculosis* and phenotypic resistance to INH, RIF, PZA, FQs and AMK that were previously selected for the construction of the TBDReaM database have been included again in our survey. Moreover, we searched the MEDLINE database for similar works issued since the TBDReaM database release (Dec 2009 – Mar 2013). We also searched for additional references in the bibliographies of the reports and reviews. Only English language articles were considered. Combinations of the following search terms were used:

Tuberculosis AND (mutation OR mutations) AND (isoniazid OR rifampin OR fluoroquinolones OR amikacin OR pyrazinamide OR katg OR maba OR fabg1 OR inha OR rpob OR rrs OR pnca OR gyra OR gyrb).

### Inclusion criteria

A study was included in the database under the following conditions: 1) it was a survey of clinical *M. tuberculosis* isolates; 2) drug sensitivity testing was performed on all isolates tested for mutations; and 3) the nucleotide or codon position and the nucleotide or amino acid change were given.

### Data retrieval

**Figure 1:**
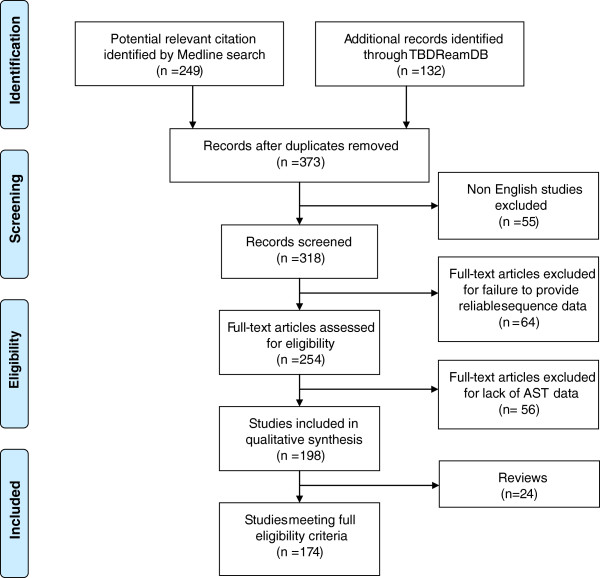
Study selection process and reasons for the exclusion of studies.

We extracted and recorded information on the following: 1) the gene in which putative resistance mutations were found and the nucleotide and/or codon position of the mutation and 2) nucleotide and/or amino acid changes. Studies that did not allow the extraction of the above data or showed discrepancies in the wild-type sequences compared with published *M. tuberculosis* genomes sequences were excluded. The workflow of the literature review is summarized in Figure 1. The extracted mutations and original citations are supplied as Additional file 1.

### Therapeutic relevance

Mutations with therapeutic relevance are tagged as “high-confidence” mutations in the MUBII-TB-DB database. High-confidence mutations have been previously defined by Sandgren *et al. *[23] as mutations associated with in vitro documented resistance reported by at least 10 publications based on the analysis of different sets of strains.

### Reference sequences

The reference sequences of the resistance genes are presented in Table 1.

**Table 1:** **List of the reference gene sequences of *Mycobacterium tuberculosis***

**Gene**	**GenBank id.**
*rpo*B	[AL123456] REGION: 759808 to 763325
*pnc*A (including the promoter)	[AL123456] REGION: 2288681 to 2289280
promoter *inh*A	[AL123456] REGION: 1673325 to 1673439
*inh*A	[AL123456] REGION: 1674202 to 1675011
*kat*G	[AL123456] REGION: 2153889 to 2156111 identical to [×68081.1]
*gyr*A	[AL123456] REGION: 7302 to 9818
*gyr*B	[AL123456] REGION: 5123 to 7267
*rrs*	[AL123456] REGION: 1471846 to 1473762 identical to [NR_102810]

### Database grammar

For each gene, there is a corresponding separate subset of the database, a text file with the description of a given mutation on each line. The line contains both the descriptions of the mutation (nucleotides and amino acids, if applicable), notes on the mutation (for instance, therapeutic relevance) and rules to apply.

The database grammar (examples are presented in Table 2) is as follows:

**Table 2:** **Example of a mutation database: a part of the *rpo*** **B mutation database for *Mycobacterium tuberculosis***

**Mutation description**	**Explanation**
A1291G ~ S431G ~ ~	Nucleotide chain: A at position 1291 is replaced by G; Protein chain: S at position 431 is replaced by G
+1300TTC ~ +434 F ~ ~	Insertion of TTC at position 1300 (F at position 434 of the protein)
CAG1306- ~ Q436- ~ ~	Deletion of CAG at position 1306 (deletion of Q at position 436 of the protein)
C1331T ~ T4441 ~ High Confidence ~ See mutDB2012	Example with a note and a remark
C1294T ~ Q432- ~ Rare ~ RULE::STOPCODON	Example with a note and a rule
CATGGACCAGAA1299- ~ MDQN434- ~ ~RULE::AMBIGUOUS= > N_ATGGACCAGAAC1300-	Example of a deletion occurring within a repeated zone. The position 1299 deduced from the nucleotide-level sequence alignment is corrected in the results

wildN*P*mutatedN ~ wildX*P*mutatedX[~Notes[~RULE::type[,action][~Remarks]]]/~Remarks]

where P is the position on the nucleotide or protein chain; N is a nucleotide (or short sequence); and X is an amino-acid or a short peptide sequence. Notes are written freely and may contain alphanumeric characters and punctuation, except the tilde sign. The specific tag “Rules::” identifies the actions to apply to clearly differentiate the rules from the remarks. Rules modify the computed result, for example, to suppress the peptide sequence if a stop codon is created or to correct the result in the case of ambiguous positions of indels that occur within repeated features. Remarks are not used by the programs but are information, such as references and dates, linked to the database entry.

Other data are also recorded in the database: the position of the first codon of the nucleotide chain, the status of the final main product (DNA/protein), and the DNA sequence of the wild strain (the reference sequence). Therefore, each gene-mutation database is a series of flat files containing the descriptions of the mutations and a series of files containing the character sequences in FASTA format as well as the locations of the final gene products and non-coding zones.

### Implementation

**Figure 2:**
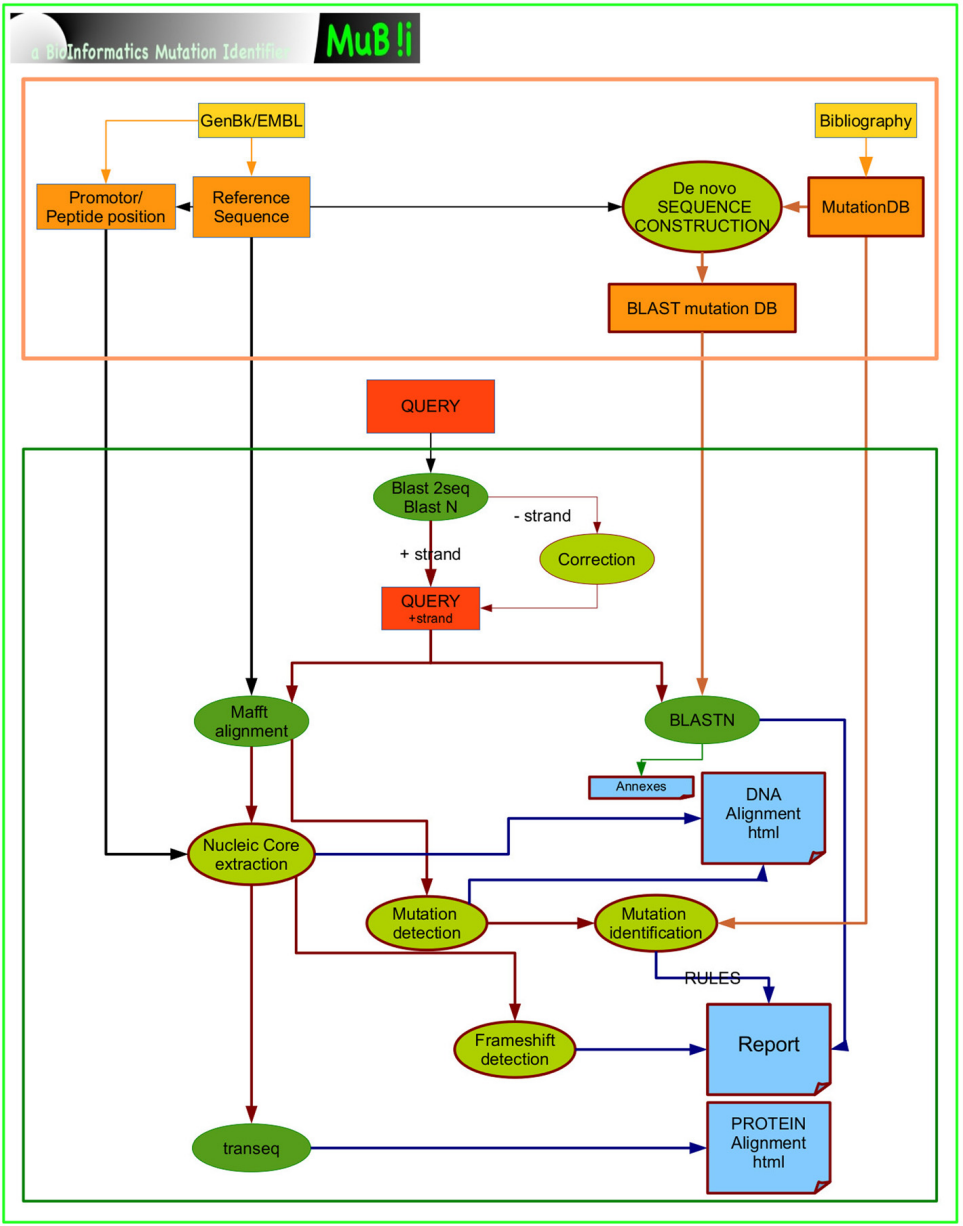
**MUBII general organization.** The MUBII-TB-DB database and the *de novo* constructed mutation database (orange frame) are built before the query session. The mutation database is used for the results interpretation and for the construction of the BLAST mutation database. Ovals indicate the use of external software (dark green) or of MUBII routines (pale green). The query analysis process (green frame) combines the BLAST result and the expertise of the alignment using the mutation database. Outputs are in blue.

MUBII is the analysis and interpretation engine and is entirely written in Python 2.7. The external programs that are called from Python are BLAST2seq [25], BLASTN, BLASTX [26] and the alignment tools MUSCLE [27] and MAFFT [28]. The routines transeq and showalign from EMBOSS [29] are also used. The scheme of the global organization is shown in Figure 2.

The Web interface exclusively uses CGI and HTML. Cascading Style Sheets are used to color the alignments. These decisions were made to achieve simplicity and reliability on every browser. The Web server runs under the APACHE MPM Prefork http server on a LINUX Debian server.

### *De novo* constructed BLASTN database

Only a few mutated sequences are available in GenBank; therefore, the construction of a database containing the sequences of all the described mutations is necessary. A sequence database is built containing a unique mutation event for each of the sequences. It contains the descriptions of the mutations present in MUBII-TB-DB, the reference sequence and the position of the promoter if required. The mutated sequences are written in the FASTA format at the nucleotide level with the description of the mutation as the descriptor. This database is rebuilt when changes occur within MUBII-TB-DB and is compiled as a BLAST database.

#### *DNA sequence analysis*

The query sequence for a given gene is submitted in the FASTA format to the program through an HTML interface. A preliminary nucleotide BLAST2seq against the gene reference sequence is used to test if the query is given as a + direct string and, if not, computes its complementary strand. BLAST2seq does not allow for the identification of mutations that may occur in the extremities of the sequence.

### Alignment to the reference and extraction of the core of the aligned sequences

The query sequence is aligned to the reference sequence by MAFFT. An algorithm identifies the core of the alignment by eliminating the trailing short sequences that are not perfectly aligned at the extremities. The core of the alignment is further used for mutation detection.

The alignment was initially produced using MUSCLE, but as the program tries to align the whole length of the reference sequence to a query that is often a partial sequence, the ends of the query were frequently assigned to positions near the ends of the reference. This problem was observed especially in the low quality-extremities of the query and has been found to occur less frequently when using MAFFT.

### Extraction of the mutations from the alignment and identification

For the core section of the alignment, a program extracts the non-matching sections and constructs a Python dictionary of the results. Each entry of this dictionary is compared with the entries of a mutation dictionary for the given gene constructed from the MUBII-TB-DB database. The resulting table contains a description of each point mutation, insertion or deletion and indicates its presence or absence in the database. In the case of deletion or insertion, the possibility of frameshift creation is also checked, and the result is added in an “alert” section. Moreover, if the mutation is known and modifies the encoded protein, this information is included. In the case of mutations usually identified by their positions in the *Escherichia coli* gene (some sections of the *rpo*B gene), the *E. coli* gene position is also computed. Finally, if the identified mutation requires interpretation, the result is corrected to fulfill the RULE:: indications. All results are saved in files in a format ready for inclusion in the HTML page.

### BLASTN on a reconstructed mutated sequence database

A BLASTN of the query against the constructed mutated sequence database is performed, and the descriptors of the best matching sequences are added to the results. The whole BLASTN result is also saved.

#### *DNA translation to protein*

The protein translation of the core of the nucleotide alignment is obtained using transeq from the EMBOSS library.

#### *Output page construction*

An HTML output file is computed for the whole alignment (DNA and protein levels) that highlights mutations using colored tags. The information gathered in the various results files is returned (detected mutations and frameshifts, mutation identification, BLAST result on the reconstructed sequence database, position within the *M. tuberculosis* wild-type gene, and, if possible, position within the *E. coli* gene). The results, alerts and alignment are placed in iframes that enable horizontal browsing and searching to inspect sequences for mutations. A specific output adapted for printing is constructed. It provides information about the detected mutations along with alignments using the showalign EMBOSS routine.

#### *Quality control*

We have used the *in silico* mutation generation routine of MUBII to generate a mutated version of the *M. tuberculosis* H37Rv sequence. For every gene and every mutation described in MUBII-TB-DB, we have constructed a sequence containing the required modification (e.g., base change, deletion, insertion). The mutated sequence thus obtained was then submitted to MUBII to verify the accuracy of the answer. All described mutations as well as hundreds of random mutations have been checked, and the MUBII results have been carefully analyzed. This procedure has identified uncertainties in mutation identification when an indel occurs near or within repeated features, especially in the *rpo*B gene. An interpretation RULE has been added to indicate such a situation and modify the answer accordingly. Following this extended quality control, both the MUBII-TB-DB database and the MUBII process have been validated for use.

## Utility and discussion

### Data input

The use of MUBII is straightforward, as the submission of the FASTA formatted sequence and the name of the corresponding gene are performed using a standard Web browser. It is also possible to use a test sequence for demonstration purposes or to verify the system.

### Data analysis

**Figure 3:**
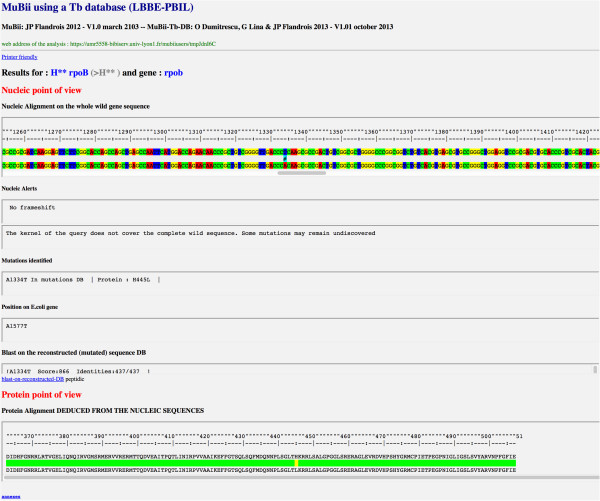
**Screen capture of an *rpo*B query: DNA alignment and detected mutations.** The figure shows an example of the detection of a mutation. The whole nucleotide alignment can be observed by horizontally scrolling the alignment window. In the case of the *rpo*B gene mutation, the nucleotide positions in *M. tuberculosis* and *E. coli* are indicated. The protein scheme compares the wild-type and query sequences for a given position. This scheme describes the changes along the protein sequence and emphasizes the effect of the mutation on the protein chain.

**Figure 4:**
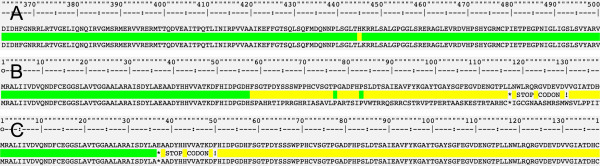
**Protein alignment and detected mutations. A** shows the detection of a mutation. **B** shows frameshift creation. **C** shows stop-codon creation. The whole alignment can be observed by horizontally scrolling the alignment window. These representations emphasize the effect of the mutation on the protein chain to provide solid understanding in terms of establishing therapy and monitoring.

The result appears in a new html page embedding the presentation of the DNA and protein alignments along with the sequence of the wild-type strain. The first section of the result pages concerns the nucleotide sequence (Figure 3). This section shows the DNA alignment of the query against the wild-type sequence and highlights any mutations. When mutations are detected, alerts are printed to note the position and the type of the mutation, its status (already described or unknown, therapeutic relevance), and situations such as frameshifts. At the end of this section, the name of the best matching sequence in the BLAST of the query against the reconstructed database of mutated sequences is shown. Access to the whole BLAST result is also possible. Because only a few strains of *M. tuberculosis* carry double mutations, the database contains only singly mutated sequences. The second section concerns the protein corresponding to the core nucleotide sequence. This section shows the peptide-level alignment of the query against the wild-type sequence and the position of mutations. The alignment is deduced from the nucleotide alignment and highlights the mutations and observed changes in the case of frameshift. In this last case, as the gaps are not introduced, the picture shows the actual changes observed in the query. This presentation is more informative, for the biologist, of the real changes that occur (Figure 4). When a stop-codon mutation is created, a specific alert is highlighted, and the shortened version of the protein is shown.

### Utility and discussion

The MUBII algorithm and the MUBII-TB-DB resistance database have been tested using amplified sequences from MTBC strains isolated in our laboratory since January 2010 (approximately 350 strains, including 30 TB-MDR strains from our collection). No mutation was detected in the MTBC strains that were classified as susceptible to the antibiotics using the in vitro susceptibility test. The mutations detected in the TB-MDR strains were fully concordant with the mutations identified in the French National Reference Centre for Mycobacteriology (National Microbiology Laboratory, Hôpital de la Pitié-Salpétrière, Paris, France). MUBII-TB-DB has been used routinely by 12 molecular biology laboratory technicians and 6 microbiologists in the Mycobacteria laboratory of the University hospital in Lyon, France for eight months. The laboratory technicians were trained to work with websites and a laboratory information management system. A very short demonstration of the use of the website was provided to each laboratory technician through the analysis of real samples. The website has been unanimously judged as user-friendly, especially because of the direct indication of mutations and the printed results that are included in the patient's record. The microbiologists who tested MUBII-TB-DB preferred its output over the previous time-consuming method combining blast2seq (at the DNA and protein levels) with searches in a local database (both electronic and paper) and/or TBDReaM database.

## Conclusions

The growing incidence and spread of antibiotic-resistant *M. tuberculosis* has led to efforts to identify the mutations in clinical isolates. The mutations linked to therapy failure are a particularly serious concern for physicians managing TB patients. The reference method is to sequence the gene of interest and then to detect and identify possible mutations. This process is time consuming, as there is no mutation database directly coupled to an analysis tool that allows for the submission of a sequence, its comparison to the database and the immediate analysis of the results. MUBII-TB-DB is a structured database collecting the description and information on modifications to seven loci of major therapeutic value in tuberculosis. The simple structure and grammar of MUBII-TB-DB contribute to the evolving capacity of the database, which can be easily updated with new entries (both mutations and resistance genes). MUBII-TB-DB is adapted to MUBII, a query and interpretation engine that is interrogated via a website. The sequence of a gene implicated in TB antibiotic resistance is analyzed simultaneously as a DNA string by BLAST against a *de novo* constructed sequence database and by an alignment process for the DNA and protein sequences. The results are graphs (alignments) and text (description of the mutation, previous description, therapeutic signification). MUBII-TB-DB is fast and easy to use, even by technicians without bioinformatics training. The system provides access to an interpretation in biological and therapeutic terms. It enables the automation of repetitive and somewhat technically challenging tasks in the tuberculosis laboratory. Its interest is to provide easy and comprehensive access to an up-to-date antibiotic resistance database concerning *M. tuberculosis*. Moreover, our algorithm is applicable to any mutation database and to any microorganism, making MUBII a useful tool for the surveillance and control of multidrug-resistant microorganisms.

## Availability

The database is available at http://umr5558-bibiserv.univ-lyon1.fr/mubii/mubii-select.cgi.

## Abbreviations

TB: Tuberculosis; WHO: World Health Organization; MDR: Multidrug Resistant; XDR: Extensively Drug Resistant; BCG: The vaccine using the attenuated “Bacille de Calmette et Guérin”; MTBC: *Mycobacterium tuberculosis* complex; RIF: Rifampicin; INH: Isoniazid; PZA: Pyrazinamide; AMK: Amikacin; FQs: Fluoroquinolones.

## Competing interests

The authors declare that they have no competing interests.

## Authors’ contributions

JPF and GL conceived the project; JPF designed the concept and implemented the system and website; OD implemented the data extraction and the resulting database; GL controlled the database; GL, OD, and JPF discussed the system during its development; OD supervised the quality assurance and the tests; GL and OD supervised the tests; JPF, OD and GL wrote the manuscript. All authors have read and approved the final manuscript.

## Supplementary Material

Additional file 1 Mutation database references spreadsheet. Click here for file
